# Predictors, Protective Factors, and Adverse Outcomes of Joint Pain among Malaysian Community-Dwelling Older Adults: Findings from the LRGS-TUA Longitudinal Study

**DOI:** 10.3390/jcm13102854

**Published:** 2024-05-12

**Authors:** Theng Choon Ooi, Nurul Fatin Malek Rivan, Suzana Shahar, Nor Fadilah Rajab, Munirah Ismail, Devinder Kaur Ajit Singh

**Affiliations:** 1 Premier Integrated Labs Sdn. Bhd., Kuala Lumpur 55100, Malaysia; thengchoon.ooi@premierintegratedlabs.com.my; 2 Centre for Healthy Ageing and Wellness, Faculty of Health Sciences, Universiti Kebangsaan Malaysia, Jalan Raja Muda Abdul Aziz, Kuala Lumpur 50300, Malaysia; fatinmalek@ukm.edu.my (N.F.M.R.); suzana.shahar@ukm.edu.my (S.S.); nfadilah@ukm.edu.my (N.F.R.); munirahismail@ukm.edu.my (M.I.)

**Keywords:** depression, disability, joint pain, predictors, older adults

## Abstract

**Background**: Joint pain has been recognized as one of the major causes of limitations in mobility, functional decline, and consequently declined quality of life in older adults. Hence, this study aimed to identify the predictors, protective factors, and adverse outcomes of joint pain in community-dwelling older adults. **Methods**: In this Long-term Research Grant Scheme—Towards Useful Ageing (LRGS-TUA) longitudinal study, a total of 1005 older participants aged 60 years and above who were successfully followed up after five years were included in the analysis. The participants self-reported their joint pain status at baseline and during the fifth year. Subsequently, the baseline characteristics were used to predict changes in joint pain status. Adverse outcomes related to joint pain were evaluated based on the participants’ joint pain statuses. **Results**: Results showed that being female, having diabetes mellitus, and higher body mass index were associated with the incidence of joint pain. Meanwhile, increased intake of pantothenic acid and higher levels of blood albumin levels were associated with recovery from joint pain. Participants with persistent joint pain at baseline and follow-up showed higher levels of depression and disability compared to individuals who never experience any joint pain. However, participants who had recovered from joint pain did not differ significantly from those without joint pain at baseline and follow-up in these measures. **Conclusions**: By identifying the modifiable risk factors, factors associated with recovery, and adverse outcomes related to joint pain, this study adds to current evidence that may contribute to further management strategies for joint pain in older adults.

## 1. Introduction

Joint pain can result from various factors, including injury, rheumatoid arthritis, gout, and osteoarthritis. Osteoarthritis is the primary cause of joint pain, particularly in older populations [1]. It not only causes pain and impairs joint function but is also a major contributor to functional decline and physical disability among older adults [2]. According to the Global Burden of Disease Study 2019, osteoarthritis ranks 18th and 21st among the leading causes of disability for individuals aged 50–74 years and 75 years and above, respectively [3]. While osteoarthritis can affect individuals of all ages, advancing age, female gender, and being overweight or obese are common risk factors associated with this degenerative condition [4].

Rapid global aging is an inevitable trend in the 21st century, leading to an anticipated increase in age-related degenerative conditions like osteoarthritis and joint pain [2,5]. While osteoarthritis can affect various joints, the knee is commonly affected, particularly in older adults [2]. In Malaysia, a multiethnic country, knee pain is reported as affecting one in every three individuals aged 55 years and above [6]. According to the Malaysian Elders Longitudinal Research (MELoR) study cohort, the prevalence of knee pain is highest among Malays (44.6%), followed by Indians (31.9%) and Chinese (23.5%), suggesting that cultural factors, lifestyle factors, and genetic variations may contribute to the differences observed [6].

Although the risk factors associated with joint pain or osteoarthritis have been reported elsewhere [4], the currently available evidence may not be applicable to the multiethnic Malaysian population. In addition, information on the adverse impact of joint pain on psychological aspects, physical and functional status, and disability among community-dwelling older adults in Malaysia is scarce. Since Malaysia is currently an aging society and is expected to become an aged nation in the year 2040, there is an urgent need to identify strategies to prevent and manage joint pain, thus reducing personal, financial, and societal burdens related to joint pain. Hence, the objective of this study was to determine the predictors of joint pain, factors associated with recovery from joint pain, and adverse outcomes of joint pain among the older participants from the Long-term Research Grant Scheme—Towards Useful Ageing (LRGS-TUA) study cohort in Malaysia who were successfully followed up after five years. The findings of this study may help bridge the gap in the existing literature by providing insights specifically tailored to the Malaysian context. This could facilitate stakeholders in addressing the complex challenges associated with joint pain among older populations in Malaysia.

## 2. Materials and Methods

### 2.1. Study Design and Participants

Data from the LRGS-TUA longitudinal study collected during baseline and five years follow-up were analyzed in this study. Ethical approval was obtained from the Medical Research and Ethics Committee of Universiti Kebangsaan Malaysia (UKM 1.5.3.5/244/NN-060-2013) and was conducted in accordance with the Declaration of Helsinki. Eligible participants who agreed to participate were required to sign the informed consent form. The study design, sampling method, and inclusion and exclusion criteria were described in detail previously [7,8]. Briefly, older adults aged 60 and above with no documented major psychiatric illness, mental disorders, or severe cognitive impairment were recruited using a multistage random sampling approach from four distinct states in Malaysia, namely, Selangor (located in the central region), Perak (in the north-west), Kelantan (in the north-east), and Johor (in the southern region). In the initial stage of sampling, one state was chosen from each geographic region. In the second stage, 35 census circles were randomly selected from each chosen state, and within these census circles, 20 living quarters were randomly chosen (third stage sampling). All eligible individuals in the selected living quarter were included in the study. Out of the 2322 participants recruited at baseline, only 1005 (43.3%) were successfully followed up after five years and included in the analysis. Participants without self-reported joint pain at baseline were examined to identify predictors associated with joint pain incidence. Meanwhile, participants with self-reported joint pain at baseline were analyzed to determine factors associated with recovery from joint pain. Adverse outcomes related to joint pain were evaluated based on the participants’ joint pain statuses during baseline and 5-year follow-up.

### 2.2. Case Definition of Joint Pain

Previous studies have indicated that joint pain can serve as a suitable surrogate for detecting osteoarthritis, particularly in large-scale population-based studies where detailed clinical examinations are challenging [6,9]. To assess the presence of joint pain at baseline and during the fifth year of follow-up, participants were asked a single question: “Are you currently experiencing joint pain?”. Those who responded “yes” were further asked if they have been diagnosed with gouty arthritis and physically examined to exclude injuries or inflammatory conditions at the affected joint. Participants with gouty arthritis or joint pain resulting from injury or inflammation were excluded from the analysis.

### 2.3. Data Collection

#### 2.3.1. Socio-Demography Data and Medical History

Participants were interviewed by trained enumerators to gather data on age, sex, ethnicity, education level, marital status, living arrangement, alcohol intake, smoking status, falls history, and self-reported medical history. Systolic and diastolic blood pressure of each participant was measured using an automated digital blood pressure monitor (Omron, Kyoto, Japan) twice to obtain the average value [7].

#### 2.3.2. Physical Measurements and Body Composition

Participants’ body mass index (BMI) was calculated using the formula “body weight (kg)/height (m)^2^”. The participants’ waist, hip, mid-upper arm, and calf circumferences were measured using a Lufkin tape, as described in detail by Shahar et al. [7]. Meanwhile, body composition was analyzed using Bio-electrical Impedance Analysis Inbody S10 (Biospace, Seoul, Republic of Korea), following the manufacturer’s guidelines.

#### 2.3.3. Psychosocial and Functional Status

The psychosocial and functional status assessment has been described previously by Shahar et al. [7]. Briefly, the depression status of the participants was determined using the validated 15-item Malay version Geriatric Depression Scale (GDS-15). The adapted 26-item Activity Lifestyle Questionnaire was used to assess the participant’s physical, mental, and social lifestyle activities. Activities of Daily Living (ADLs), Instrumental Activity of Daily Living (IADL), and World Health Organization Disability Assessment Schedule 2.0 (WHODAS 2.0) were used to assess self-care function, independent living skills, and disability, respectively. Meanwhile, the Medical Outcome Social Support (MOSS) survey was used to measure functional social support. The feelings of loneliness, perception of stress, self-perceived success, life satisfaction, and personality disorder were evaluated using the three-item Loneliness Scale, four-item Perceived Stress Scale, eight-item Flourishing Scale, Satisfaction with Life Scale, and Eysenck Personality Questionnaire, respectively.

#### 2.3.4. Assessment of Dietary Intake

Participants’ weekly food and drink intakes were recorded using a validated Dietary Habits Questionnaire specialized for older adults [10]. Then, the Nutritionist Pro^TM^ Software version 2.2.1 (Axxya Systems, Stafford, TX, USA) was used to analyze the daily nutrient intake profile of each participant based on their respective dietary record.

#### 2.3.5. Physical Performance

The participant’s physical fitness was assessed using the Senior Fitness Test battery (2 min step test, chair stand test, chair sit-and-reach test, dominant handgrip strength test, and back scratch test) and timed up and go (TUG) test, which were used to evaluate aerobic endurance, lower limb muscle strength, lower body flexibility, upper limb muscle strength, upper body flexibility, as well as mobility and balance status, respectively [7]. Meanwhile, the Malay version Physical Activity Scale for the Elderly (PASE) questionnaire was used to assess older adults’ physical activity over seven days in 3 different domains, namely, leisure, household, and work-related activity [11].

#### 2.3.6. Cognitive Function Assessments

The MMSE and Montreal Cognitive Assessment (MoCA) tests were used to determine the participants’ global cognitive function. The working memory and psychomotor performance were evaluated using the Digit Span Test and Digit Symbol Test, respectively. Then, the Rey Auditory Verbal Learning Test (RAVLT) test was used to test the short-term verbal memory of the participants. Detailed methodology on how to conduct the neurocognitive assessment was described previously [7].

#### 2.3.7. Blood Samples Collection and Biochemical Analysis

Fasted venous blood (overnight fasting of at least 8 h) was collected from each participant by a trained phlebotomist, as described previously [12]. Then, the blood samples were sent to an accredited clinical diagnostic laboratory for biochemical analysis.

### 2.4. Statistical Analysis

The incidence rate of joint pain was calculated using a person-year analysis. A statistical analysis was conducted using the Statistical Package for the Social Sciences version 25.0 (IBM Corp, Armonk, NY, USA). A descriptive analysis was conducted to study the baseline parameters. Independent *t*-tests and chi-square tests were used for univariate comparisons of continuous and categorical data between the groups, respectively. Then, significant variables (*p* < 0.05) were subjected to univariate binary logistic regression analysis first before being entered into the final multivariate binary logistic regression model. The model was adjusted for potential confounding factors (age, living status, smoking status, alcohol drinking status, falls history, and medications associated with joint pain), and all the significance variables (*p* < 0.05) in the analysis were identified as predictors of joint pain or factors associated with recovery from joint pain. To assess the impact of joint pain on various health outcomes, one-way analysis of variance (ANOVA), followed by Tukey post hoc analysis, was performed.

## 3. Results

### 3.1. The Prevalence and Incidence Rate of Joint Pain among Older Adults

**Figure 1:**
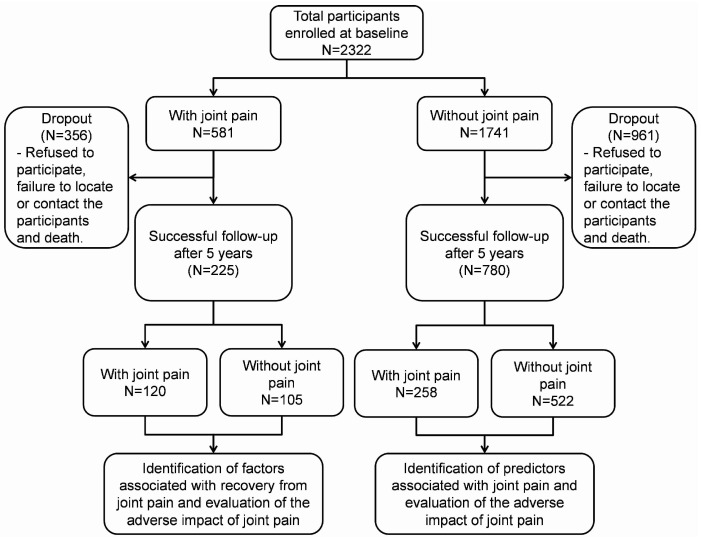
Illustration of the number of participants from the baseline to the 5-year follow-up.

As shown in Figure 1, 2322 participants were recruited at baseline, with 581 (25.0%) reporting joint pain. After five years of follow-up, the prevalence of joint pain increased to 37.6%. The incidence rate of joint pain among community-dwelling older adults in Malaysia was 6.62 cases per 100 person years. Table 1 presents the baseline characteristics of participants categorized by their self-reported joint pain status at baseline and five years’ follow-up.

### 3.2. Predictors of Joint Pain among Older Adults

Out of the 1741 participants without joint pain at baseline, 780 (44.80%) were successfully followed up. Among them, 258 self-reported experiencing joint pain during the follow-up period. These participants, compared to those without joint pain, were more likely to be female, be single/divorced/widowed, have lower years of formal education, be diagnosed with hypertension and diabetes, have higher BMI, hip circumference, fat mass, body fat percentage, and lower skeletal muscle mass, perform poorer in chair stand and dominant handgrip tests, score lower in MoCA and digit symbol tests, and have lower IADL scores (Table 1). The univariate binary logistic regression analysis of significant variables is presented in Table S1. After adjusting for potential confounding factors, being female [odd ratio (OR): 1.479; 95% confidence interval (CI): 1.067–2.049], having diabetes (OR: 1.467; 95% CI: 1.011–2.129), and a higher BMI (OR: 1.053; 95% CI: 1.012–1.096) were predictors of joint pain among Malaysian community-dwelling older adults (Table 2).

### 3.3. Factors Associated with Recovery from Joint Pain in Older Adults

Out of the 581 participants who reported having joint pain at baseline, 225 (38.73%) were successfully followed up. Among them, 105 either recovered or no longer reported joint pain during the follow-up period. They were more likely to have higher years of formal education, higher intake of pantothenic acid, higher albumin levels, have better performance in the 2 min step and TUG tests, compared to participants who continued to have joint pain (Table 1). The univariate binary logistic regression analysis of significant variables is presented in Table S2. After adjusting for potential confounding factors, higher intake of pantothenic acid (OR: 3.661; 95% CI: 1.097–13.102) and higher albumin levels (OR: 1.199; 95% CI: 1.021–1.408) were associated with recovery from joint pain among Malaysian community-dwelling older adults (Table 3).

### 3.4. Impact of Joint Pain on Various Health-Related Outcomes among Older Adults

The impact of different joint pain statuses on disability levels and psychological, physical, and functional status were examined after a five-year follow-up. Significant differences (*p* < 0.05) were found in the GDS-15 score, lifestyle activities, WHODAS 2.0, and PASE scores among participants with different joint pain statuses (Table 4). The Tukey post hoc analysis revealed that participants with joint pain at baseline and during the five-year follow-up had higher GDS-15 and WHODAS 2.0 scores (*p* < 0.05), as well as lower lifestyle activities and PASE scores compared to those without joint pain. Participants without joint pain at baseline but with joint pain during the follow-up had higher WHODAS 2.0 scores and lower lifestyle activities compared to those without joint pain throughout the study. Notably, participants who recovered from joint pain showed improvements in all outcomes, with no significant differences compared to those without joint pain throughout the study, suggesting that the deficits associated with joint pain are reversible.

## 4. Discussion

The prevalence of knee pain at baseline and five years’ follow-up was 25.0% and 37.6%, respectively, indicating an increased risk with age. This is consistent with osteoarthritis, the primary cause of joint pain in older adults, which involves joint structural tissue degradation over time [1]. Additionally, being female is also a predictor of joint pain symptoms, aligning with previous findings that reported a higher incidence of osteoarthritis in women after the age of 50, as compared to men [1].This may be attributed to a series of changes in body composition, such as weight gain and muscle loss, following menopause [13,14]. These changes have been associated with an increased risk of having osteoarthritis [15,16]. In addition, variation in societal function and activities may also contribute to the gender differences in joint pain prevalence [17].

Being overweight or obese is a significant risk factor for knee osteoarthritis development [16,18]. Increased body weight places additional mechanical stress on the weight-bearing knee joint, thus augmenting the risk of joint tissue degradation [16]. Moreover, obesity is associated with metabolic and physiological changes that favor the pro-inflammatory state, hence indirectly contributing to osteoarthritis [1,19,20]. Additionally, obesity is a predisposing factor for diabetes mellitus, which was identified as one of the predictors of knee pain in this study [21]. It is speculated that hyperglycemic conditions in diabetes may stimulate oxidant formation and accelerate the breakdown and degeneration of the cartilage matrix, thus increasing the likelihood of osteoarthritis [22,23].

The factors that promote recovery from joint pain were also identified in this study. Older adults who recovered from joint pain had higher blood albumin levels and increased intake of pantothenic acids. Albumin, a protein synthesized by the liver and present in the blood plasma, plays a role in fluid retention and facilitates the transport of various substances in the body, including vitamins, enzymes, and hormones [24]. A previous study has shown that patients with rheumatoid arthritis have lower albumin levels compared to healthy individuals [25]. Since low albumin levels may be associated with inflammatory conditions or infections, higher albumin levels could indicate the resolution of inflammation, thereby alleviating symptoms of osteoarthritis such as pain [26].

Pantothenic acid, also known as vitamin B5, plays a crucial role as a metabolic precursor for coenzyme A. Previous studies have demonstrated that patients with rheumatoid arthritis have lower blood pantothenic acid levels than healthy individuals, and daily injection of 50 mg calcium pantothenate or intake of calcium pantothenate (<2 g/d) can relieve the symptoms of rheumatoid arthritis [27,28]. However, the validity of these previous findings needs further validation as they were conducted a long time ago. Moreover, no evidence supports the use of pantothenic acid in the treatment of other forms of arthritis. Hence, future studies are needed to confirm the potential protective effects of pantothenic acid in alleviating knee pain.

According to the Global Burden of Disease Study 2019, osteoarthritis is the 18th and 21st cause of disability among individuals aged 50–74 years and 75 years and above, respectively [3]. The disability associated with joint pain is most likely due to increased limitations in performing daily activities and mobility, thus posing burdens with significant implications for the affected individuals [29]. In fact, our findings demonstrate that older adults with joint pain are more physically inactive and engage in fewer lifestyle activities. This can be attributed to pain symptoms and other associated symptoms like stiffness, swelling, and loss of flexibility due to joint deformities [30]. Although joint pain may hinder physical activity, exercise is crucial for improving muscle strength around the affected joints [31,32]. Furthermore, accumulating evidence suggests that various exercise trainings can prevent cartilage degeneration, inhibit inflammation, and prevent loss of the subchondral bone and metaphyseal bone trabeculae, thus relieving pain, stiffness, joint dysfunction, and muscle weakness in people with knee osteoarthritis [31,32].

Concerning depressive symptoms, a meta-analysis has revealed that one in five individuals with osteoarthritis experiences depressive symptoms [33]. Recent studies have suggested that higher pain levels, decreased function, multiple affected joints, and slower gait speed may contribute to depression in people with osteoarthritis [34]. The causes of depression in osteoarthritis are multifaceted, involving common risk factors like obesity, as well as interactions between psychosocial factors (social isolation, catastrophizing, perceived discrimination, stress), biological mechanisms (inflammation, neuroendocrine dysregulation), and other associated factors [17,34]. The co-existence of depression in people with osteoarthritis may require a biopsychosocial model of care [17].

This study has some limitations. While joint pain is often considered a suitable surrogate of osteoarthritis in population-based studies, our study was unable to completely rule out other potential causes of joint pain as it is challenging to conduct detailed clinical examinations in a community setting with a large sample size [6,9]. However, individuals with gouty arthritis and joint pain resulting from injury or inflammation, as confirmed through physical examinations, were excluded from the study. Additionally, the self-reported joint pain symptoms were not restricted to specific joint sites. However, most joint pain symptoms due to osteoarthritis were reported to occur at the weight-bearing joints such as knees and hips. Despite these limitations, this nationwide population-based study with a large sample size provides valuable insights into predictors, protective factors, and the impact of joint pain among older adults over a five-year period.

In conclusion, being female, having diabetes mellitus, and higher BMI appeared to be predictors of joint pain in older Malaysians. Conversely, higher blood albumin levels and daily intake of pantothenic acid were associated with recovery from joint pain. Furthermore, our findings revealed that older adults experiencing chronic joint pain exhibited greater disability, depressive symptoms, reduced physical activity, and lower functional status compared to their healthy counterparts. However, it is noteworthy that these negative outcomes have the potential to be mitigated upon recovery from joint pain. These insights contribute valuable knowledge to the field, emphasizing the importance of addressing joint pain and its associated factors for promoting better health outcomes and quality of life among the rapidly growing older populations in Malaysia. Moving forward, it is essential to consider the identified modifiable risk and protective factors, along with the adverse impacts, when developing prevention and management strategies for joint pain in the older population.

## Figures and Tables

**Table 1:** The characteristics of the participants aged 60 years and above from the baseline of the LRGS-TUA study, overall and by self-reported joint pain status at baseline and five years’ follow-up.

	*n* (%) or Mean ± SD							
Baseline	Without Joint Pain during Baseline				With Joint Pain during Baseline			
Five Years Follow-Up	Total *n* = 780 (100)	Without Joint Pain *n* = 522 (66.9)	With Joint Pain *n* = 258 (33.1)	*p*-Value	Total *n* = 225 (100)	Without Joint Pain *n* = 105 (46.7)	With Joint Pain *n* = 120 (53.3)	*p*-Value
Age	67.81 ± 5.33	67.78 ± 5.35	67.86 ± 5.32	0.854	67.89 ± 5.48	67.78 ± 5.78	67.99 ± 5.22	0.774
Sex								
Male	388 (49.7)	282 (54.0)	106 (41.1)	0.001 **	90 (40.0)	42 (40.0)	48 (40.0)	1.000
Female	392 (50.3)	240 (46.0)	152 (58.9)		135 (60.0)	63 (60.0)	72 (60.0)	
Ethnicity								
Malay	475 (60.9)	309 (59.2)	166 (64.3)	0.349	136 (60.4)	62 (59.0)	74 (61.7)	0.718
Chinese	275 (35.3)	191 (36.6)	84 (32.6)		74 (32.9)	37 (35.2)	37 (30.8)	
Indian	30 (3.8)	22 (4.2)	8 (3.1)		15 (6.7)	6 (5.7)	9 (7.5)	
Marital status								
Married	580 (74.4)	400 (76.6)	180 (69.8)	0.039 *	156 (69.3)	74 (70.5)	82 (68.3)	0.728
Single/divorced/widowed	200 (25.6)	122 (23.4)	78 (30.2)		69 (30.7)	31 (29.5)	38 (31.7)	
Staying								
With others	713 (91.4)	484 (92.7)	229 (88.8)	0.063	201 (89.3)	95 (90.5)	106 (88.3)	0.603
Alone	67 (8.6)	38 (7.3)	29 (11.2)		24 (10.7)	10 (9.5)	14 (11.7)	
Education (years)	5.67 ± 4.11	5.88 ± 4.21	5.24 ± 3.88	0.039 *	4.98 ± 3.86	5.54 ± 4.17	4.48 ± 3.52	0.040 *
Smoking	131 (16.8)	89 (17.0)	42 (16.3)	0.786	33 (14.7)	13 (12.4)	20 (16.7)	0.365
Drinking alcohol	36 (4.6)	26 (5.0)	10 (3.9)	0.489	8 (3.6)	2 (1.9)	6 (5.0)	0.211
Falls history	144 (18.5)	92 (17.6)	52 (20.2)	0.391	52 (23.1)	19 (18.1)	33 (27.5)	0.095
Medical history								
Hypertension	371 (41.6)	232 (44.4)	139 (53.9)	0.013 *	113 (50.2)	49 (46.7)	64 (53.3)	0.318
Hypercholesterolemia	221 (28.3)	159 (30.5)	62 (24.0)	0.061	89 (39.6)	40 (38.1)	49 (40.8)	0.675
Cardiovascular disease	65 (8.3)	44 (8.4)	21 (8.1)	0.890	24 (10.7)	11 (10.5)	13 (10.8)	0.931
Diabetes mellitus	178 (22.8)	106 (20.3)	72 (27.9)	0.017 *	54 (24.0)	22 (21.0)	32 (26.7)	0.317
Physical measurement								
BMI (kg/m^2^)	24.95 ± 4.14	24.60 ± 4.10	25.65 ± 4.14	0.001 **	25.84 ± 4.08	25.31 ± 4.41	26.29 ± 3.74	0.072
Waist circumference (cm)	87.58 ± 10.47	87.24 ± 10.47	88.26 ± 10.44	0.201	89.85 ± 11.00	89.37 ± 11.47	90.27 ± 10.60	0.542
Hip circumference (cm)	96.12 ± 8.63	95.52 ± 8.30	97.34 ± 9.15	0.006 **	98.80 ± 9.41	97.83 ± 9.44	99.65 ± 9.34	0.148
Calf circumference (cm)	33.50 ± 3.59	33.39 ± 3.67	33.73 ± 3.41	0.214	33.84 ± 3.61	33.64 ± 3.96	34.02 ± 3.29	0.438
Fat mass (kg)	24.13 ± 9.00	23.46 ± 8.90	25.50 ± 9.06	0.003 **	26.38 ± 8.88	25.49 ± 9.19	27.16 ± 8.55	0.162
Fat-free mass (kg)	36.87 ± 7.64	37.24 ± 7.83	36.12 ± 7.20	0.055	36.16 ± 8.13	36.44 ± 8.50	35.92 ± 7.82	0.635
Skeletal muscle mass (kg)	19.61 ± 4.56	19.84 ± 4.68	19.14 ± 4.27	0.040 *	19.15 ± 4.85	19.34 ± 5.06	18.98 ± 4.66	0.570
Percentage of body fat (%)	38.83 ± 10.35	37.91 ± 10.25	40.70 ± 10.31	<0.001 ***	41.67 ± 10.00	40.52 ± 9.94	42.67 ± 9.99	0.107
Systolic (mmHg)	138.44 ± 19.29	138.39 ± 19.62	138.56 ± 18.65	0.908	139.51 ± 22.35	140.80 ± 21.77	138.44 ± 22.86	0.445
Diastolic (mmHg)	76.34 ± 12.33	76.45 ± 12.46	76.11 ± 12.10	0.729	77.85 ± 13.14	78.79 ± 13.38	77.08 ± 12.94	0.344
Nutrition								
Energy (kcal/day)	1678.48 ± 473.75	1685.93 ± 474.76	1663.45 ± 472.31	0.546	1642.33 ± 506.74	1680.76 ± 496.27	1608.09 ± 515.72	0.303
Protein (g/day)	71.15 ± 21.06	70.93 ± 20.64	71.60 ± 21.94	0.682	71.17 ± 25.09	71.93 ± 23.59	70.50 ± 26.44	0.684
Carbohydrate (g/day)	228.47 ± 79.00	229.65 ± 79.86	226.08 ± 77.33	0.564	220.31 ± 76.40	228.43 ± 79.35	213.08 ± 73.28	0.149
Fat (g/day)	52.91 ± 19.29	53.30 ± 19.11	52.12 ± 19.65	0.436	52.99 ± 20.59	53.16 ± 19.44	52.85 ± 21.65	0.914
Vitamin A (µg/day)	1258.66 ± 800.14	1244.69 ± 802.48	1286.82 ± 796.29	0.503	1223.16 ± 815.35	1202.51 ± 713.19	1241.55 ± 889.52	0.731
Vitamin C (mg/day)	123.87 ± 86.70	125.57 ± 82.38	120.44 ± 94.90	0.452	112.37 ± 85.22	110.42 ± 84.37	114.10 ± 86.33	0.757
Vitamin D (mg/day)	0.27 ± 0.84	0.27 ± 0.74	0.28 ± 1.00	0.858	0.31 ± 1.00	0.40 ± 1.30	0.23 ± 0.61	0.252
Vitamin E (mg/day)	10.70 ± 49.83	11.03 ± 53.00	10.03 ± 42.81	0.798	10.79 ± 63.30	4.82 ± 3.21	16.12 ± 86.83	0.176
α-tocopherol (mg/day)	0.48 ± 1.29	0.48 ± 1.24	0.48 ± 1.40	0.982	0.45 ± 1.21	0.45 ± 1.35	0.44 ± 1.09	0.971
Thiamin (mg/day)	1.36 ± 3.19	1.25 ± 2.90	1.58 ± 3.71	0.220	1.71 ± 3.80	1.88 ± 3.52	1.55 ± 4.05	0.537
Riboflavin (mg/day)	1.24 ± 0.48	1.25 ± 0.48	1.22 ± 0.47	0.429	1.21 ± 0.50	1.23 ± 0.47	1.20 ± 0.53	0.677
Niacin (mg/day)	10.51 ± 4.02	10.47 ± 3.87	10.57 ± 4.32	0.769	10.38 ± 4.32	10.62 ± 3.91	10.18 ± 4.67	0.463
Pyridoxine (mg/day)	0.73 ± 0.37	0.73 ± 0.37	0.73 ± 0.37	0.823	0.69 ± 0.33	0.70 ± 0.35	0.68 ± 0.32	0.559
Folate (µg/day)	106.06 ± 69.77	105.76 ± 64.08	106.65 ± 80.18	0.871	104.35 ± 85.03	110.02 ± 86.62	99.29 ± 83.66	0.365
Cobalamin (µg/day)	4.02 ± 3.73	4.05 ± 3.81	3.94 ± 3.56	0.709	3.75 ± 3.83	3.51 ± 3.72	3.97 ± 3.93	0.385
Pantothenic Acid (mg/day)	0.28 ± 0.44	0.29 ± 0.44	0.26 ± 0.45	0.348	0.27 ± 0.42	0.34 ± 0.52	0.21 ± 0.30	0.030 *
Vitamin K (µg/day)	16.47 ± 67.91	17.55 ± 69.12	14.28 ± 65.50	0.540	21.69 ± 71.46	22.04 ± 64.89	21.37 ± 77.14	0.946
Sodium (mg/day)	1499.45 ± 1037.74	1500.83 ± 1004.78	1496.66 ± 1103.35	0.959	1469.00 ± 949.18	1505.88 ± 825.52	1436.14 ± 1049.85	0.598
Potassium (mg/day)	1538.38 ± 525.78	1548.37 ± 515.30	1518.24 ± 546.85	0.466	1500.63 ± 610.97	1514.19 ± 615.07	1488.54 ± 609.85	0.763
Calcium (mg/day)	519.21 ± 229.99	516.84 ± 223.26	523.98 ± 243.39	0.693	536.39 ± 263.67	568.62 ± 307.42	507.68 ± 214.85	0.103
Iron (mg/day)	13.73 ± 5.20	13.85 ± 5.29	13.49 ± 5.03	0.389	13.54 ± 5.49	13.65 ± 5.19	13.44 ± 5.76	0.782
Phosphorus (mg/day)	1107.40 ± 393.98	1099.07 ± 386.50	1124.20 ± 408.95	0.417	1112.80 ± 476.23	1124.30 ± 473.68	1102.56 ± 480.42	0.743
Magnesium (mg/day)	133.49 ± 65.39	133.45 ± 63.85	133.56 ± 68.52	0.982	127.16 ± 63.51	128.37 ± 66.89	126.08 ± 60.64	0.796
Zinc (mg/day)	3.72 ± 1.96	3.70 ± 1.87	3.78 ± 2.13	0.604	3.53 ± 1.74	3.72 ± 1.87	3.37 ± 1.60	0.141
Selenium (µg/day)	24.70 ± 18.31	24.82 ± 18.43	24.46 ± 18.09	0.802	24.28 ± 18.05	26.32 ± 17.78	22.46 ± 18.18	0.125
Copper (mg/day)	0.61 ± 0.36	0.61 ± 0.36	0.59 ± 0.34	0.319	0.56 ± 0.32	0.57 ± 0.34	0.54 ± 0.30	0.484
Manganese (mg/day)	0.44 ± 0.59	0.45 ± 0.60	0.42 ± 0.59	0.485	0.45 ± 0.51	0.49 ± 0.55	0.41 ± 0.47	0.285
Molybdenum (µg/day)	0.39 ± 1.64	0.42 ± 1.78	0.33 ± 1.33	0.487	0.35 ± 1.74	0.39 ± 1.99	0.31 ± 1.49	0.720
Biochemical								
Hemoglobin (g/L)	13.99 ± 2.09	14.08 ± 2.14	13.80 ± 1.97	0.110	14.23 ± 2.19	14.44 ± 2.54	14.04 ± 1.83	0.240
Fasting blood glucose (mmol/L)	5.97 ± 1.93	5.97 ± 2.00	5.97 ± 1.79	0.970	6.03 ± 1.66	5.82 ± 1.30	6.22 ± 1.89	0.099
Total cholesterol (mmol/L)	5.38 ± 1.09	5.39 ± 1.11	5.37 ± 1.03	0.824	5.40 ± 1.14	5.45 ± 1.17	5.36 ± 1.11	0.621
HDL cholesterol (mmol/L)	1.41 ± 0.35	1.39 ± 0.35	1.44 ± 0.34	0.106	1.39 ± 0.37	1.43 ± 0.41	1.36 ± 0.32	0.180
LDL cholesterol (mmol/L)	3.31 ± 0.99	3.33 ± 1.02	3.26 ± 0.93	0.364	3.35 ± 1.05	3.31 ± 1.12	3.38 ± 1.00	0.682
Triglyceride (mmol/L)	1.49 ± 0.76	1.48 ± 0.77	1.50 ± 0.75	0.862	1.45 ± 0.47	1.55 ± 0.73	1.38 ± 0.61	0.094
Albumin (g/L)	42.91 ± 2.52	42.95 ± 2.56	42.81 ± 2.45	0.517	42.78 ± 2.39	43.49 ± 2.42	42.18 ± 2.21	<0.001 ***
Physical Performance								
2 min step test (number)	65.53 ± 23.95	66.43 ± 23.93	63.69 ± 23.93	0.139	62.25 ± 25.93	66.84 ± 25.82	58.14 ± 25.45	0.013 *
Chair stand test (number)	10.53 ± 3.07	10.72 ± 3.11	10.16 ± 2.95	0.019 *	9.92 ± 2.66	10.25 ± 2.61	9.62 ± 2.67	0.078
Chair sit-and-reach (cm)	0.97 ± 11.39	1.06 ± 11.39	0.79 ± 11.41	0.758	0.29 ± 10.64	0.85 ± 9.97	−0.22 ± 11.24	0.462
Timed up and go Test (seconds)	10.86 ± 3.90	10.69 ± 3.51	11.21 ± 4.59	0.084	11.27 ± 2.99	10.68 ± 2.23	11.80 ± 3.46	0.005 **
Dominant handgrip strength test (kg)	24.14 ± 7.82	24.60 ± 7.91	23.21 ± 7.58	0.023 *	22.73 ± 7.69	23.15 ± 6.94	22.35 ± 8.31	0.444
Back scratch test (cm)	14.05 ± 13.16	13.42 ± 13.02	15.33 ± 13.39	0.062	16.44 ± 12.82	16.25 ± 14.40	16.61 ± 11.30	0.841
Cognitive function								
MMSE	23.75 ± 4.35	23.85 ± 4.34	23.55 ± 4.35	0.353	23.24 ± 4.65	23.64 ± 4.24	22.90 ± 4.97	0.237
MoCA	19.65 ± 5.52	19.97 ± 5.39	19.00 ± 5.75	0.022 *	19.12 ± 5.37	19.68 ± 5.51	18.63 ± 5.22	0.149
Digit symbol	5.31 ± 2.67	5.46 ± 2.81	4.99 ± 2.32	0.017 *	5.11 ± 2.67	5.33 ± 2.52	4.92 ± 2.79	0.281
Digit span	7.69 ± 2.32	7.75 ± 2.36	7.57 ± 2.25	0.312	7.57 ± 2.61	7.69 ± 2.83	7.47 ± 2.40	0.528
RAVLT T5 score	38.73 ± 10.41	38.84 ± 10.51	38.50 ± 10.22	0.679	38.4 ± 10.06	39.26 ± 10.31	37.66 ± 9.83	0.253
Psychosocial & functional status								
ADL	6.00 ± 0.04	6.00 ± 0.00	6.00 ± 0.06	0.318	6.00 ± 0.00	6.00 ± 0.00	6.00 ± 0.00	-
IADL	12.75 ± 1.95	12.87 ± 1.84	12.49 ± 2.13	0.016 *	12.66 ± 1.90	12.73 ± 1.84	12.61 ± 1.95	0.645
WHODAS 2.0	6.11 ± 8.96	5.70 ± 8.96	6.94 ± 8.91	0.074	7.10 ± 8.41	6.34 ± 7.98	7.76 ± 8.75	0.216
Satisfaction with Life Scale	8.25 ± 2.19	8.28 ± 2.17	8.19 ± 2.24	0.612	7.98 ± 2.48	8.27 ± 2.19	7.73 ± 2.68	0.104
Eysenck Personality Questionnaire	2.01 ± 2.88	2.00 ± 2.93	2.03 ± 2.78	0.887	2.61 ± 3.36	2.16 ± 3.14	2.99 ± 3.51	0.065
Loneliness Scale	3.22 ± 0.84	3.20 ± 0.77	3.26 ± 0.96	0.344	3.33 ± 1.07	3.23 ± 0.84	3.42 ± 1.22	0.166
Flourishing Scale	13.50 ± 6.34	13.47 ± 6.64	13.55 ± 5.68	0.859	14.75 ± 7.80	14.64 ± 7.05	14.85 ± 8.41	0.846
Perceived Stress Scale	2.97 ± 2.84	3.05 ± 2.89	2.80 ± 2.74	0.251	3.16 ± 3.01	3.08 ± 2.85	3.23 ± 3.14	0.720
MOSS Survey	39.38 ± 14.78	39.74 ± 14.89	38.67 ± 14.55	0.343	38.48 ± 15.56	38.23 ± 14.86	38.70 ± 16.20	0.822
Geriatric Depression Scale	2.45 ± 2.19	2.38 ± 2.22	2.58 ± 2.15	0.236	2.90 ± 2.25	2.67 ± 1.98	3.11 ± 2.46	0.148

Note: Data were presented as mean ± SD or *n* (%). Univariate comparison between the groups performed using Independent *t*-test and Chi-square test for continuous and categorical data, respectively. * *p* < 0.05; ** *p* < 0.01; *** *p* < 0.001. Abbreviation: ADL, Activities of Daily Living; BMI, body mass index; HDL, high-density lipoprotein; IADL; Instrumental Activities of Daily Living; LDL, low-density lipoprotein; MMSE, Mini-Mental State Examination; MoCA, Montreal Cognitive Assessment; MOSS, Medical Outcome Social Support; RAVLT, Rey Auditory Verbal Learning Test; WHODAS 2.0, World Health Organization Disability Assessment Schedule 2.0.

**Table 2:** The predictors associated with self-reported joint pain among community-dwelling older adults.

Risk Factors Category	Item	Self-Reported Joint Pain Status		
		*p*-Value	OR	[95% CI]
Sociodemographic	Sex			
	Male	Reference	Reference	Reference
	Female	0.019 *	1.479	1.067–2.049
Medical history	Diabetes mellitus			
	No	Reference	Reference	Reference
	Yes	0.044 *	1.467	1.011–2.129
Physical measurement	BMI	0.011 *	1.053	1.012–1.096

Note: The final multivariate binary logistic regression model was adjusted with age, living status, smoking status, alcohol drinking status and falls history. * *p* < 0.05. Abbreviation: BMI, body mass index; CI, confidence interval; OR, odd ratio.

**Table 3:** The factors associated with recovery from self-reported joint pain among community-dwelling older adults.

Protective Factors Category	Item	Self-Reported Joint Pain Status		
		*p*-Value	OR	[95% CI]
Nutrition	Pantothenic acid	0.049 *	3.661	1.097–13.102
Biochemical	Albumin	0.027 *	1.199	1.021–1.408

Note: The final multivariate binary logistic regression model was adjusted with age, living status, smoking status, alcohol drinking status, falls history and the use of medications associated with joint pain. * *p* < 0.05. Abbreviation: CI, confidence interval; OR, odd ratio.

**Table 4:** Outcome analysis based on the status of joint pain at baseline and five years’ follow-up.

Outcomes	Mean ± SD				*p*-Value
Baseline	Without Joint Pain (*n* = 780)		With Joint Pain (*n* = 225)		
Five Years Follow-Up	Without Joint Pain (*n* = 522)	With Joint Pain (*n* = 258)	Without Joint Pain (*n* = 105)	With Joint Pain (*n* = 120)	
GDS-15 score	2.82 ± 2.51	3.16 ± 2.51	2.62 ± 2.23	3.69 ± 2.69 ^ab^	0.002 **
Lifestyle activities	40.77 ± 11.77	38.14 ± 11.91 ^a^	39.02 ± 12.08	36.63 ± 11.84 ^a^	0.001 **
WHODAS 2.0	4.33 ± 5.35	6.90 ± 6.73 ^a^	5.18 ± 6.20	8.56 ± 6.42 ^ab^	<0.001 ***
PASE	117.03 ± 61.26	110.72 ± 58.57	112.28 ± 58.83	96.35 ± 55.21 ^a^	0.008 **

Note: Data were presented as mean ± SD or *n* (%). The impact of different joint pain statuses on various health outcomes were performed using the one-way ANOVA followed by Tukey posthoc analysis. ** *p* < 0.01; *** *p* < 0.001. ^a^ Significant different (*p* < 0.05) compared to participants without self-reported joint pain during baseline and five years follow-up. ^b^ Significant different (*p* < 0.05) compared to participants with self-reported joint pain during baseline but recovered during five years follow-up. Abbreviation: GDS-15, 15 items Malay version Geriatric Depression Scale; PASE, Physical Activity Scale for the Elderly; WHODAS 2.0, World Health Organization Disability Assessment Schedule 2.0.

## Data Availability

The data that support the findings of this study are available from the corresponding author upon reasonable request.

## Supplementary Materials

The following supporting information can be downloaded at: https://www.mdpi.com/article/10.3390/jcm13102854/s1, Table S1: The univariate scores for individual predictors of self-reported joint pain.; Table S2: The univariate scores for individual factors associated with recovery from self-reported joint pain.
