# Supplementary material for: Predictors, Protective Factors, and Adverse Outcomes of Joint Pain among Malaysian Community-Dwelling Older Adults: Findings from the LRGS-TUA Longitudinal Study
Source: J Clin Med. 2024 May 12;13(10):2854. doi: 10.3390/jcm13102854 (PMC11122189; doi:10.3390/jcm13102854)
Supplement: Supplementary file 1 [file jcm-13-02854-s001.zip › jcm-2988963-supplementary.pdf]

Table S1. The univariate scores for individual predictors of self-reported joint pain.

Risk Factors Category	Item	Self-Reported Joint Pain Status		
		p-value	OR	[95 % CI]
**Sociodemographic**	**Sex**			
	- Male	**Reference**	**Reference**	**Reference**
	- Female	**0.001****	1.685	1.246 – 2.279
	**Marital status**			
	- Married	**Reference**	**Reference**	**Reference**
	- Single/divorced/widowed	**0.039***	1.421	1.017 – 1.985
	**Education**	**0.040***	0.962	0.927 – 0.998
**Medical history**	**Hypertension**			
	- No	**Reference**	**Reference**	**Reference**
	- Yes	**0.013***	1.460	1.082 – 1.970
	**Diabetes mellitus**			
	- No	**Reference**	**Reference**	**Reference**
	- Yes	**0.018***	1.519	1.075 – 2.146
**Physical measurement**	**BMI**	**0.001****	1.063	1.025 – 1.102
	**Hip circumference**	**0.006****	1.025	1.007 – 1.043
	**Fat mass**	**0.003****	1.025	1.008 – 1.043
	**Skeletal muscle mass**	**0.047***	0.966	0.934 – 1.000
	**Percentage body fat**	**0.001****	1.027	1.012 – 1.042
**Physical Performance**	**Chair stand test**	**0.020***	0.941	0.894 – 0.990
	**Dominant handgrip strength test**	**0.023***	0.977	0.958 – 0.997
**Cognitive function**	**MoCA**	**0.022***	0.969	0.943 – 0.996
	**Digit symbol**	**0.026***	0.933	0.877 – 0.992
**Psychosocial & functional status**	**IADL**	**0.012***	0.909	0.844 – 0.979

Note: The data were analysed using univariate binary logistic regression analysis. * p<0.05; ** p<0.01.
Abbreviation: BMI, body mass index; CI, confidence interval; IADL; Instrumental Activities of Daily Living; MoCA, Montreal Cognitive Assessment; OR, odd ratio.

Table S2. The univariate scores for individual factors associated with recovery from self-reported joint pain.

Protective Factors Category	Item	Self-Reported Joint Pain Status		
		p-value	OR	[95 % CI]
**Sociodemographic**	**Education**	**0.041***	1.075	1.003 – 1.152
**Nutrition**	**Pantothenic acid**	**0.037***	2.350	1.053 – 5.246
**Biochemical**	**Albumin**	**0.001****	1.297	1.120 – 1.502
**Physical Performance**	**2-minute step test**	**0.015***	1.014	1.003 – 1.025
	**TUG test**	**0.007****	0.865	0.778 – 0.961

Note: The data were analysed using univariate binary logistic regression analysis. * p<0.05; ** p<0.01.
Abbreviation: CI, confidence interval; OR, odd ratio; TUG, timed up and go.
